# Limbic oxytocin receptor expression alters molecular signaling and social avoidance behavior in female prairie voles (*Microtus ochrogaster*)

**DOI:** 10.3389/fnins.2024.1409316

**Published:** 2024-07-16

**Authors:** Lina K. Nerio-Morales, Arjen J. Boender, Larry J. Young, Marisol R. Lamprea, Adam S. Smith

**Affiliations:** ^1^Department of Pharmacology and Toxicology, School of Pharmacy, University of Kansas, Lawrence, KS, United States; ^2^Department of Psychiatry and Behavioral Sciences, Center for Translational Social Neuroscience, Yerkes National Primate Research Center, Emory University, Atlanta, GA, United States; ^3^Department of Psychology, School of Human Sciences, Universidad Nacional de Colombia, Bogotá, Colombia; ^4^Program in Neuroscience, School of Pharmacy, University of Kansas, Lawrence, KS, United States

**Keywords:** oxytocin receptor, social defeat stress, prairie vole (*Microtus ochrogaster*), nucleus accumbens, anterior cingulate, basolateral amygdala, female

## Abstract

**Introduction:**

The social defeat paradigm is the most representative animal model to study social anxiety disorder (SAD) and its underlying neuronal mechanisms. We have previously reported that defeat progressively reduces oxytocin receptors (OXTR) in limbic regions of the brain over an eight-week period in female prairie voles (*Microtus ochrogaster*). Oxytocin receptors activate the mitogen-activated protein kinase (MAPK) pathway, which has been previously associated with the anxiolytic effects of oxytocin. Here, we assessed the functional significance of OXTR in stress-induced social avoidance and the response of the MAPK signaling pathway in the nucleus accumbens (NAc), anterior cingulate cortex (ACC), and basolateral amygdala (BLA) of female prairie voles.

**Methods:**

In experiment 1, Sexually naïve adult female prairie voles were defeated for three consecutive days and tested a week after for social preference/avoidance (SPA) test. Control subjects were similarly handled without defeat conditioning. In experiment 2, sexually and stress naïve adult female prairie voles were bilaterally injected into the NAc, ACC, or the BLA with a CRISPR/Cas9 virus targeting the Oxtr coding sequence to induce OXTR knockdown. Two weeks post-surgery, subjects were tested for SPA behavior. Viral control groups were similarly handled but injected with a control virus. A subgroup of animals from each condition in both experiments were similarly treated and euthanized without being tested for SPA behavior. Brains were harvested for OXTR autoradiography, western blot analysis of MAPK proteins and quantification of local oxytocin content in the NAc, BLA, ACC, and PVN through ELISA.

**Results:**

Social defeat reduced OXTR binding in the NAc and affected MAPK pathway activity and oxytocin availability. These results were region-specific and sensitive to exposure to the SPA test. Additionally, OXTR knockdown in the NAc, ACC, and BLA induced social avoidance and decreased basal MAPK activity in the NAc. Finally, we found that OXTR knockdown in these regions was associated with less availability of oxytocin in the PVN.

**Conclusion:**

Dysregulation of the oxytocin system and MAPK signaling pathway in the NAc, ACC, and BLA are important in social behavior disruptions in female voles. This dysregulation could, therefore, play an important role in the etiology of SAD in women.

## Introduction

Social anxiety disorder (SAD) is characterized by generalized social avoidance and marked anxiety symptoms in social situations (Stein and Stein, 2008). SAD is the second most common anxiety disorder, with 12.1% prevalence in the United States and 4.4% worldwide (Stein et al., 2017). This condition has a mean duration of 20 years (Kessler et al., 1994) and occurs more in women than men (Stein et al., 2017). Research suggests that biological sex influences an individual’s stress response, brain functioning and structure, and development of SAD and other anxiety disorders (Asher et al., 2017). It is worth noting that social gender factors and identity are also indicators of the manifestation and duration of anxiety disorders (see Moscovitch et al., 2005; Bekker and van Mens-Verhulst, 2007; Christiansen, 2015). Chronic exposure to social stress or social conflict and peer victimization (e.g., bullying and humiliation) promotes the onset of SAD, resulting in functional impairments in the individual (Muller et al., 2005; Gren-Landell et al., 2011; Aderka et al., 2012). In addition, these factors contribute to the development of other psychiatric conditions, including major depressive disorder and substance use disorder (Moutier, 1999). While SAD treatment involves cognitive behavioral therapy and pharmacological treatment, including antidepressants and anticonvulsants (Blanco et al., 2002; Garakani et al., 2020), not all patients respond to these treatments (Bruce et al., 2005). Therefore, the mechanisms driving the pathophysiology of SAD and its high prevalence in women warrant further investigation.

Social defeat has been extensively used as one of the most representative animal model to study stress-induced social avoidance and the underlying neuronal mechanisms in different rodent species (Toth et al., 2012; Toth and Neumann, 2013; Kuske and Trainor, 2022). Defeat involves repeated exposure to aggression from other conspecifics, resulting in psychological stress (Kudryavtseva et al., 1991; Hollis and Kabbaj, 2014). The monogamous prairie vole (*Microtus ochrogaster*) has been validated as an animal model to study SAD (Smith et al., 2013; Tickerhoof et al., 2020; Hale et al., 2021). Once defeated, male and female prairie voles display social avoidance of strangers and anxiety-like behaviors (Tickerhoof et al., 2020), characteristic traits in human SAD (Stein and Stein, 2008). Previous findings using this animal model showed sexual disparities in neurobiology sensitive to stress. While males and females receive similar defeat experiences and develop avoidant behavior (Tickerhoof et al., 2020), defeated female prairie voles show a progressive reduction of oxytocin receptor (OXTR) binding in mesocorticolimbic regions (Tickerhoof et al., 2020), as observed in other defeat animal models (Duque-Wilckens et al., 2018; Wang L. et al., 2018). Conversely, male prairie voles show a progressive reduction of serotonin 1A receptor binding in the basolateral amygdala (BLA) and the dorsal raphe nucleus after defeat (Hale et al., 2021). Further, the social deficits induced by defeat are recovered after intranasal treatment with oxytocin only in defeated females (Duque-Wilckens et al., 2018; Hale et al., 2021), highlighting the anxiolytic effects of the neuropeptide and its relevance in the etiology of SAD in this sex.

The anxiolytic effects of oxytocin have been reported in different animal models. Oxytocin injections in the PVN of female prairie voles before stress exposure resulted in reduced anxiety-like behaviors and hypothalamic-pituitary-adrenal (HPA) axis activity (Smith et al., 2016). Moreover, microinjections of oxytocin in other brain regions involved in social behavior facilitation have also been associated with anxiolysis (Blume et al., 2008; Yoshida et al., 2009; Wang L. et al., 2018), an effect that is reversed by the administration of OXTR antagonists (Wang L. et al., 2018). These behavioral effects have been attributed to the activation of the MAPK pathway. For instance, administration of oxytocin in the PVN of male and female Wistar rats increases MAPK phosphorylation and decreases anxiety-like behaviors (Blume et al., 2008; Jurek et al., 2012). This is particularly relevant as the MAPK pathway responds to different stress models, including social defeat stress (Meller et al., 2003; Pardon et al., 2005; Iio et al., 2011). While defeat induces changes in OXTR density in limbic regions, there is no direct evidence that OXTR signaling and the MAPK pathway in these regions mediate the social deficits observed in defeated females.

In this study, we investigated the role of the oxytocin system and the MAPK pathway in defeat-induced social avoidance in female prairie voles as an animal model to study SAD. We first explored the effects of social defeat on the oxytocin system and the activation state of the MAPK pathway. In a second set of experiments, we induced a knockdown of OXTR using a Clustered Regularly Interspaced Short Palindrominc Repeats/Cas9 (CRISPR/Cas9) viral construct in the nucleus accumbens (NAc), anterior cingulate cortex (ACC), and BLA to assess the effects on social preference/avoidance (SPA) behavior and the activity of the MAPK signaling pathway. In these experiments, we included a subgroup of animals not exposed to the SPA test to determine whether the phosphorylation state of MAPK proteins is also sensitive to a behavioral test involving social novelty. This allowed us to determine changes in MAPK response to a social challenge in control-conditioned and treated animals. We hypothesized that social defeat and OXTR downregulation cause social avoidance and impair the activation of the MAPK signaling pathway in limbic structures of female prairie voles.

## Materials and methods

### Animals

Subjects were laboratory-bred sexually naïve female prairie voles derived from an Illinois wild-caught population. The voles were weaned on postnatal day 21 ± 3 and housed with a same-sex conspecific in cages (29.2 L × 19.1 W × 12.7 H cm). Access to food (Teklad Global Rabbit Diet 2030) and water was available *ad libitum* in a room maintained at a temperature range of 21 ± 1°C and a 14:10 light:dark cycle with lights on at 06:00.

Residents were female prairie voles paired with a vasectomized male and previously screened as aggressors. Briefly, intruder female prairie voles were exposed to a different resident vole each day for 5 min. Only female residents displaying at least three offensive attacks against intruders throughout three consecutive days were classified as aggressive. Residents displaying latching attacks were classified as hyperaggressive and excluded from the study. The voles were inspected for injuries and closely monitored throughout the test to ensure that neither animal was seriously harmed. All behavioral tests were performed between 13:00–17:00 (social preference/avoidance test) or 09:00–12:00 (all other tests). All procedures were conducted following the National Institutes of Health Guide for the Care and Use of Laboratory Animals and were approved by the Animal Care and Use Committee of The University of Kansas in Lawrence, KS. An effort was made to minimize the number of animals used and their suffering.

### Behavior tests

#### Social defeat

The social defeat protocol was performed as previously described with some adjustments (Tickerhoof et al., 2020). Briefly, residents and intruders were transferred to the behavioral testing rooms to acclimate for at least 30 min. After habituation, the resident’s cage mate, nesting material, water bottle, and food hopper were removed from the cage. Female residents were left to acclimate in the empty cage for an additional 15 min prior to defeat conditioning. The subject vole (i.e., the female intruder) was placed into the aggressor’s (i.e., resident’s) cage to allow for interaction for 15 min or seven resident-initiated attacks, whatever happened first. Subjects were then rapidly screened for injuries and placed into a small stainless-steel corral (9 L × 9 W × 14 H cm) in the center of the resident’s cage for the rest of the test (1 h total) to allow intruder exposure to the olfactory cues and threat from the resident. Subjects were exposed to a different aggressor each of the three consecutive days of defeat conditioning. One subject was immediately removed from the study due to severe injuries. Control animals received no defeat conditioning and were handled similarly. Briefly, control subjects were transported to the behavioral rooms for at least 30 min habituation and transferred to empty cages for 1 h throughout the 3 days of control conditioning. Animals were returned to their home cage and transferred to the colony room.

#### Social preference/avoidance test

The social preference/avoidance (SPA) test was performed as previously described (Tickerhoof et al., 2020). Briefly, the subject vole was placed in the center of a three-chamber arena containing a thin layer of corn cob bedding and allowed to explore for 10 min. Then, the subject vole was secluded in the central chamber with barriers. One stainless-steel corral was placed in each of the remaining chambers: one corral housed an unfamiliar female prairie vole, and the other an unfamiliar object, namely a black rubber stopper (i.e., non-social stimulus). The barriers were removed, and the subject was allowed to explore the arena for 10 min. The subject’s location was recorded from above and manually scored using JWatcher (UCLA) to analyze active investigation (i.e., sniffing), proximity to stimulus, and cage crosses defined as crosses from one chamber to the next.

### Viral vector design and infusion

An adeno-associated viral vector (AAV) expressing CRISPR/Cas9 was used to induce OXTR knockdown. The AAV9-CRISPR/Cas9 viral construct was used to target the prairie vole *Oxtr* coding sequence as previously described (Boender and Young, 2020). The viral tools used consist of a cocktail containing either AAV9-U6-sgRNA(pan-Oxtr1)-CMV-eGFP (1.5 × 10^10^ genomic copies/μL) or the control vector, AAV9-U6-sgRNA (LacZ)-CMV-eGFP (1.5 × 10^10^ genomic copies/μL), mixed with the viral vector expressing the Cas9 protein, AAV9-RSV-spCas9 (1.5 × 10^10^ genomic copies/μL), hereinafter called knockdown cocktail and control cocktail, respectively. The viral constructs were developed and provided by Dr. Boender and Dr. Young from Emory University in Atlanta, GA. The knockdown cocktail reduces a ~60% of OXTR binding after 6 weeks of incubation (Boender and Young, 2020). From our previous studies, defeat causes about 30% decrease in OXTR binding in the NAC, ACC and the BLA after a week of stress conditioning (Hale et al., 2021). For this experiment, we reduced the viral incubation to two weeks to induce similar downregulation of OXTR receptors in the NAc, ACC, and BLA. AAV infusions were performed in adult female prairie voles under ketamine (75 mg/kg) and dexmedetomidine (1 mg/kg) anesthesia in a stereotaxic frame (Stoelting Co., Wood Dale, IL) with a heating pad for thermoregulation. Female voles were also administered subcutaneously meloxicam (2 mg/kg) for analgesia and lidocaine (2 mg/kg) for local anesthesia. Subjects received intracranial bilateral injections with 300 nL of the knockdown or control cocktail in either NAc, BLA, or ACC (see coordinates in Supplementary Table S1) using a 30-gage 2 μL blunt Hamilton syringe at an infusion rate of 1 nL/s. The syringe was left in place for 5 min following injection to minimize diffusion up the needle track. Following AAV injections, voles were returned to the colony room and housed for 2 weeks until behavioral testing and brain extraction.

### Biological characterization

#### Brain collection, processing, and protein quantification

Brains were sectioned with a cryostat at 20 microns for receptor autoradiography analysis and 200 and 300 microns for ELISA and western blot analysis of the NAc, ACC, and BLA. Additional 300-micron sections were collected for ELISA analysis of the PVN. Coronal sections of rostral regions (i.e., NAc and ACC) started at approximately +1.70 mm from bregma and continued in sequence. The collection of caudal regions (i.e., PVN and BLA) started at approximately −0.58 mm from bregma and continued in sequence as described in Supplementary Table S2. Bilateral tissue punches of 1 mm were collected from each region except for the PVN, which was dissected through a single micro punch per brain section. Tissue punches were extracted via sonication with either 40 μL (NAc, BLA and ACC) or 15 μL (PVN) of ice-cold RIPA extraction buffer (10 mM Trizma Base; 150 mM NaCl; 1 mM EDTA; 0.1% SDS; 1% Triton X-100; 1% Sodium Deoxycholate) containing 0.1% protease and phosphatase inhibitor cocktail (Halt, Thermo Scientific, cat#78441). Lysates were centrifuged at 18,800 × g for 20 min at 4°C to remove cellular debris. Protein concentration was determined via BCA assay according to the manufacturer’s instructions (Pierce, Thermo Scientific, cat#23246). Protein extractions and slides for autoradiography were stored at −80°C until further analysis.

#### Western blots

Protein extractions from the NAc, ACC, and BLA containing 15 μg of total protein were separated on 12% Tris-Glycine gels (Novex, Thermo Fisher Scientific, cat#WXP01226BOX) and transferred into a nitrocellulose membrane (BIO-RAD cat#1620115). Samples across treatment groups were semi-randomly organized within gels. Following electrophoresis and transfer, membranes were stained with 5% Ponceau S staining to measure total protein content. After the stain was removed with distilled deionized water, membranes were blocked with 5% bovine serum albumin (BSA) in Tris-buffered saline containing 0.1% Tween 20 (0.1% TBS-T) and incubated overnight at 4°C with the primary antibody of the anti-phosphoprotein (Supplementary Table S3). Membranes were then incubated for 1 h at room temperature with the appropriate horseradish peroxidase (HRP)-conjugated secondary antibody (Supplementary Table S3). Membrane signal development was performed after a 10-min incubation with luminol (SuperSignal^™^ West Dura Extended Duration Substate; Thermo Scientific, cat# 34076). The chemiluminescent signal was analyzed with the ChemiDoc^™^ Imaging System after signal development. Following image acquisition, membranes were washed with 0.1% TBS-T, and the HRP signal was quenched with 10% acetic acid for 30 min at 37°C. After HRP quenching, membranes were washed with 0.1% TBS-T, blocked with non-fat dry milk in TBS-T, and re-probed with the anti-protein studied. Protein band intensity was processed by densitometric analysis using ImageJ software (Rasband, W.S., ImageJ, U. S. National Institutes of Health, Bethesda, Maryland, United States, https://imagej.nih.gov/ij/, 1997–2018) for Experiment 1, and GelAnalyzer 19.1 (available at www.gelanalyzer.com) by Istvan Lazar Jr., PhD and Istvan Lazar Sr., PhD, CSc, for Experiment 2. Results for each sample were expressed as relative densitometry after normalizing against Ponceau S staining and expressed as the ratio of the phosphorylated and non-phosphorylated form of the proteins for each region independently.

#### Receptor autoradiography

Receptor binding was used to analyze differences in the expression of the OXTR as previously described (Hale et al., 2021). Coronal 20 μm sections containing NAc, ACC, and BLA were pre-washed twice in 170 mM Tris–HCl, pH 7.4 for 5 min at room temperature, and fixed with 0.5% formaldehyde in 100 mM phosphate buffer solution for 5 min at room temperature. Slides were then washed with binding solution (160 mM Tris–HCl; 10 mM MgCl_2_; 0.10% BSA; 0.05% Bacitracin; pH 7.4) for 5 min and incubated for 1 h at room temperature in a binding solution containing 50 pM ^125^I-Ornithine Vasotocin Analog (OVTA; Perkin Elmer, Cat# NEX 254). Following incubation, slides were washed twice in ice-cold binding solution for 5 min, rinsed with washing buffer (50 mM Tris–HCl; 100 mM MgCl_2_, pH 7.4) under stirring for 35 min at room temperature and briefly dipped in deionized water. Slides were left overnight to air dry and exposed to autoradiography film (Carestream Health Biomax MR; Fisher Scientific, cat# 8952855) for 8 days protected from light. Development was performed using the medical film processor SRX-101A and scanned for further image processing. Autoradiograms were analyzed using ImageJ software (Rasband, W.S., ImageJ, US National Institutes of Health, Bethesda, MD, United States, http://imagej.nih.gov/ij/). The optical density of OXTR binding for the NAc, ACC, and BLA was measured using at least two brain sections for each region per brain and averaged across sections. The background was measured from adjacent areas with no binding and subtracted from the readings for all brain regions.

#### Oxytocin ELISA

Oxytocin levels in extracted micro punches were measured using the Oxytocin Enzyme Immunoassay kit (Enzo Life Sciences, Farmingdale, NY, Cat# ADI-900-153) according to the manufacturer’s instructions. The kit is highly specific for oxytocin and has a minimum assay sensitivity of 15 pg./mL. ELISA for NAc, ACC, and BLA was run using 10 μL of sample and spiked with 90 μL of an oxytocin standard solution (100 pg./mL). Extracts from PVN were run using 5 μL of sample and 95 μL of standard buffer. Absorbance was measured using a Synergy Biotek plate reader, and total oxytocin concentration was determined according to the kit’s instructions. After subtraction of the spiking solution, when applicable, and normalization against the total protein of the sample, the results were expressed as picograms of oxytocin per microgram of total protein.

### Experimental design

#### Experiment 1—social defeat regulation of oxytocin system and signaling proteins

**Figure 1:**
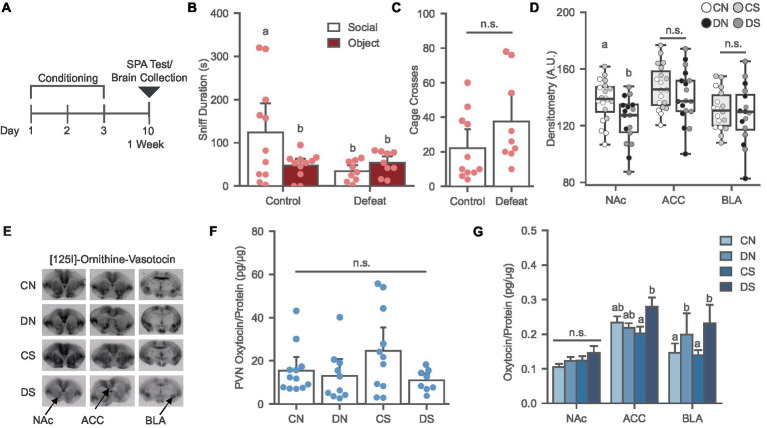
Social defeat causes social avoidance and decreases oxytocin binding in the NAc of female prairie voles. **(A)** Timeline for Experiment 1 examining the effects of social defeat and the SPA test on oxytocin system. **(B)** Social defeat induces social avoidance phenotype. Different letters indicate a significant difference vs. control social. **(C)** Cage cross frequency was similar across conditions (*N* = Control: 11, Defeat: 9). **(D)** Social defeat reduces OXTR binding in the NAc. Letters indicate significant difference from values with no shared letters within region (*N* = 16–20 per condition). A.U., Arbitrary Unit. **(E)** Ornithine Vasotocin Analog, [125]-OVTA sample images of OXTR autoradiography. **(F)** PVN oxytocin levels was similar across conditions (*N* = 8–11 per condition). **(G)** Oxytocin levels increased in the ACC of defeated animals after SPA test and social defeat trend to increase BLA oxytocin. Letters above bars significantly differ from values with no shared letters within region (*N* = 8–12 per condition). CN, controls not exposed to the SPA test; DN defeated subjects not exposed to the SPA test; CS, controls exposed to the SPA test; DS, defeated subjects exposed to the SPA test.

Adult female prairie voles (70–120 days old) were exposed to social defeat conditioning for three consecutive days and split into two cohorts. The first cohort was tested for social SPA behavior 1 week after defeat conditioning and sacrificed 20 min later via rapid decapitation (Figure 1A). The second cohort was likewise handled but without being tested for SPA. Control subjects were similarly grouped but received no defeat conditioning. Subjects not tested for SPA were transported 1 week after defeat or control conditioning to the behavioral room for acclimation and sacrificed as described earlier. A total of four groups were studied: controls exposed to the SPA test (CS; *n* = 11), controls not exposed to the SPA test (CN; *n* = 12), defeated subjects exposed to the SPA test (DS; *n* = 9) and defeated subjects not exposed to the SPA test (DN; *n* = 10). The brains were harvested, flash frozen on dry ice, and stored at −80°C until further processing.

#### Experiment 2—oxytocin receptor regulation of social avoidance and signaling proteins

Adult female prairie voles (60–120 days old) were injected bilaterally with 300 nL per side of an AAV CRISPR viral knockdown cocktail to induce regional knockdown of OXTR (Boender and Young, 2020). Animals were injected in one of the three regions of interest, namely, NAc, ACC, and BLA. Voles were split into two cohorts per region. Two weeks later after stereotactic surgery, the first cohort of animals was tested for SPA and euthanized 20 min later via rapid decapitation. The second cohort was likewise handled without being tested for SPA. Control subjects were similarly grouped but were injected bilaterally with 300 nL per side of the control AAV CRISPR viral cocktail into the NAc, ACC, or BLA. Control injection into different target regions had no effect on SPA behavior and behavioral data from these animals were pooled, following the practice from previous studies (Lim and Young, 2004; Namburi et al., 2015; Li and Hollis, 2021). Subjects not tested for SPA were transported to the behavioral room two weeks after stereotactic surgery for acclimation and sacrificed. The NAc, ACC, BLA, and PVN were punched from all control groups regardless of injection site for biological characterization. A total of four groups were studied: viral controls exposed to the SPA test (VCS; ACC, *n* = 4; BLA, *n* = 3; NAc, *n* = 3), viral controls not exposed to the SPA test (VCN; ACC, *n* = 3; BLA, *n* = 3; NAc, *n* = 3), knockdown subjects exposed to the SPA test (KDS; ACC, *n* = 11; BLA, *n* = 8; NAc, *n* = 13), and knockdown subjects not exposed to the SPA test (KDN; ACC, *n* = 9; BLA, *n* = 10; NAc, *n* = 10). The brains were harvested, flash frozen on dry ice, and stored at −80°C until further processing.

### Data analysis

Statistical analysis was performed using SPSS 28 (IBM) after the exclusion of outliers, defined as two standard deviations above or below the mean (Berger and Kiefer, 2021). Final sample sizes after outlier exclusion are detailed in the respective figure caption. Different analyses of variance (ANOVA) were used based on the experimental design. Appropriate parametric or non-parametric tests were performed to evaluate statistical significance after checking for normality of the data. Simple main effects or interactions were reported followed by pair wise comparisons with Tukey HSD. Dunn’s procedure was performed for pair-wise comparisons in non-parametric data after a significant Kruskal-Wallis test. Data are presented as mean + standard error of the mean (SEM), and statistical significance was set at *p* < 0.05. We performed a mixed-design ANOVA [Injected site: NAc, ACC, vs. BLA (between subjects) × stimuli: social vs. object (within-subjects)] to determine differences in sniff duration during SPA behavior resulting from viral control injection site in the NAc vs. ACC vs. BLA. The effect of the injection site on sniff duration in the viral controls was not significant with a small effect size [*F* (2, 8) = 0.244, *p* = 0.789, η^2^ = 0.058], indicating that injection site in control subjects did not affect the results on SPA behavior. Thus, voles injected with the control virus regardless of injection site were pooled into a single control condition for all other analyzes.

Data from the SPA test was examined using 2 × 2 mixed factorial experimental design. For Experiment 1: Condition (control, defeat, between-subjects) x stimuli (social, object, within-subjects). For Experiment 2: Treatment (viral control, viral knockdown, between-subjects) x stimulus (social, object, within-subjects).

Data from cage crosses was analyzed using a single factor between-subjects design in both experiments: Experiment 1 (control, defeat) and Experiment 2 (viral control, NAc OXTR knockdown, ACC OXTR knockdown, and BLA OXTR knockdown).

Data from receptor autoradiography, ELISA, and MAPK signaling markers was analyzed for each region using a 2 × 2 factorial design. For Experiment 1: Condition (control, defeat) × behavior (No SPA, SPA). For Experiment 2: Treatment (viral control, viral knockdown) × behavior (No SPA, SPA).

## Results

### Experiment 1—social defeat regulation of oxytocin system and signaling proteins

#### Social defeat induces social avoidance in female prairie voles and decreases OXTR binding in the NAc

Social defeat conditioning led to a significant reduction of social preference relative to controls in the SPA-tested groups [Figure 1B; *F* (1, 18) = 4.457, *p* = 0.049] without affecting the novel object investigation or general exploration of the arena [Figure 1C; *F* (1, 18) = 2.344, *p* = 0.143].

SPA testing did not affect OXTR binding in the NAc, ACC, and BLA (Figures 1D,E). A main effect of defeat was observed in the NAc [Figure 1D; *F* (1, 33) = 8.066, *p* = 0.008], where defeat conditioning significantly reduced OXTR binding by up to 36%, as previously reported (Wang L. et al., 2018; Hale et al., 2021). However, this effect was not observed in the ACC [Figure 1D; *F* (1, 33) = 1.296, *p* = 0.263] or BLA [Figure 1D; *F* (1, 30) = 0.330, *p* = 0.570], revealing a robust sensitivity of NAc to defeat conditioning.

#### Social defeat induces region specific effects on local oxytocin levels

The effects of social defeat and SPA testing on PVN, NAc, ACC, and BLA oxytocin levels were examined. No significant chances in oxytocin levels were observed in the PVN after defeat and SPA [Figure 1F; H (3) = 4.984, *p* = 0.173]. Two-way ANOVA revealed a significant interaction between the SPA test and defeat conditioning in the ACC [Figure 1G; *F* (1, 37) = 5.248, *p* = 0.028], where the defeat group exposed to SPA had higher oxytocin levels than the defeat group not exposed to SPA, indicating increased local oxytocin in defeated animals after the behavioral test. In the BLA, a trend was observed in the main effect of defeat [Figure 1G; *F* (1, 35) = 3.056, *p* = 0.089], where defeat increased the levels of the neuropeptide compared to the control groups. There were no defeat or SPA test effects in the NAc oxytocin levels [Figure 1G; *F* (1, 36) = 0.031, *p* = 0.862].

#### Social defeat induces region specific effects on MAPK signaling

**Figure 2:**
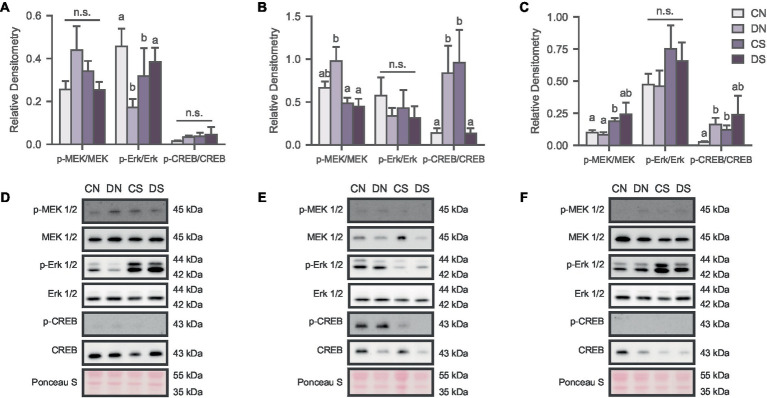
Social defeat induces stimuli- and region-specific changes in the activation state of the MAPK pathway. Activity of the signaling pathway expressed as a ratio of the phosphorylated and non-phosphorylated densitometry of the markers in **(A)** NAc; **(B)** ACC; and **(C)** BLA. Letters above bars significantly differ from values with no shared letters within each marker (*N* = 6–12 per condition). Representative blots for MAPK markers in **(D)** NAc; **(E)** ACC; and **(F)** BLA. CN, controls not exposed to the SPA test; DN defeated subjects not exposed to the SPA test; CS, controls exposed to the SPA test; DS, defeated subjects exposed to the SPA test.

To determine whether defeat conditioning and SPA test altered MAPK activation in the NAc, ACC, and BLA, we analyzed the phosphorylation state of MEK 1/2 (*p*-MEK 1/2), Erk 1/2 (*p-*Erk 1/2) and CREB (*p*-CREB), relative to total protein content. Our results revealed several region-specific effects that were dependent on defeat, SPA testing and specific combinations of the two (Figure 2). In the NAc, two-way ANOVA revealed an interaction between the behavioral test and defeat conditioning in *p*-Erk/Erk ratio [Figure 2A; H (3) = 8.549, *p* = 0.036] where SPA test decreased Erk 1/2 phosphorylation in control animals while having the opposite effect in defeated subjects. No significant differences were observed for MEK 1/2 [Figure 2A; H (3) = 2.419, *p* = 0.490] and CREB activity [Figure 2A; *F* (1, 33) = 0.085, *p* = 0.773].

MEK 1/2 phosphorylation was significantly reduced in defeated animals exposed to SPA in the ACC [Figure 2B; H (3) = 8.380, *p* = 0.039]. A similar effect was observed for the *p*-CREB/CREB ratio [Figure 2B; H (3) = 11.882, *p* = 0.008], indicating hyperactivation of MAPK signaling proteins in the resting state of defeated animals but decreased activity after the behavioral test. The opposite effect was observed in the control groups with increased phosphorylation of CREB after SPA testing (Figure 2C). There were no significant differences in the activation state of Erk 1/2 phosphorylation across groups [Figure 2B; *F* (1, 35) = 0.036, *p* = 0.113].

MAPK phosphorylation in the BLA was affected by defeat and SPA testing, as observed in the activation state of MEK 1/2 [Figure 2C; H (3) = 8.000, *p* = 0.046] and partially in the *p*-CREB/CREB ratio [Figure 2C; H (3) = 8.398, *p* = 0.038], where defeated subjects showed increased phosphorylation of these markers.

### Experiment 2—oxytocin receptor regulation of social avoidance and signaling proteins

#### Oxytocin receptor knockdown in the NAc, ACC, and BLA induces social avoidance in female prairie voles

**Figure 3:**
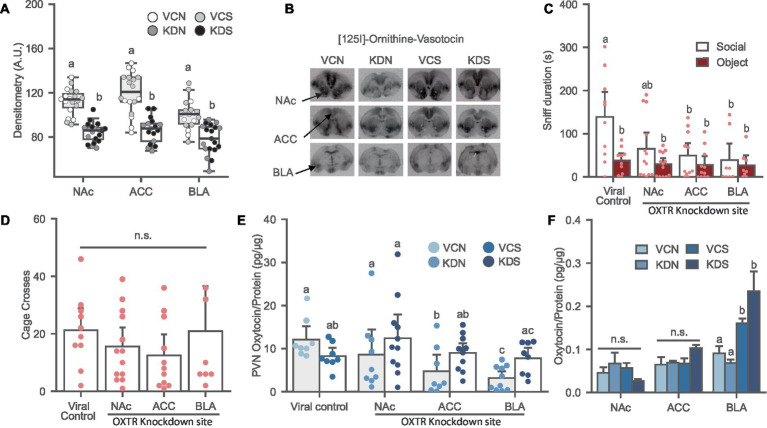
Oxytocin receptor knockdown in the NAc, ACC and BLA induces social avoidance and affects local oxytocin levels in the PVN of female prairie voles. **(A)** Oxytocin receptor binding decreased after OXTR knockdown in the NAc, ACC, and BLA of stress-naïve female prairie voles. Letters above bars indicate significant difference within region (*N* = 17–20 per condition). A.U., Arbitrary Unit. **(B)** Ornithine Vasotocin Analog, [125]-OVTA sample images of OXTR autoradiography. **(C)** Oxytocin receptor knockdown in the NAc, ACC, and BLA prevents social preference. Letters indicate a significant difference vs. viral control social. **(D)** Cage cross frequency was similar across treatment groups (*N* = Viral control: 10, NAc: 13; ACC: 11; BLA: 7). **(E)** Oxytocin receptor knockdown decreased oxytocin levels in the PVN of BLA and the ACC knockdown groups not exposed to SPA test. Letters above bars significantly differ from values with no shared letters within knockdown region vs. viral control groups (*N* = 8–11 per condition). **(F)** SPA test increased oxytocin levels in the BLA regardless of viral treatment. Letters above bars significantly differ within region (*N* = NAc: 5–13; ACC: 8–11; BLA: 8–10, per condition). VCN, viral controls not exposed to the SPA test; VCS, viral controls exposed to the SPA test; KDN Knockdown subjects not exposed to the SPA test; KDS Knockdown subjects exposed to the SPA test.

Oxytocin receptor knockdown was validated using receptor autoradiography. Subjects treated with the knockdown cocktail had a significant reduction of OXTR binding in the NAc [*F* (1, 35) = 65.831, *p* ≤ 0.001], ACC [*F* (1, 31) = 41.388, *p* ≤ 0.001], and BLA [*F* (1, 33) = 23.277, *p* ≤ 0.001], reaching up to 40% downregulation of the receptor across regions (Figures 3A,B).

Oxytocin receptor knockdown in the ACC and BLA induced social avoidance while a trend was observed in the NAc treated group relative to the viral control [Figure 3C; *F* (1, 38) = 2.451, *p* = 0.078]. Overall, OXTR knockdown eliminated social preference across all groups without affecting the novel object investigation and general exploration of the arena [Figure 3D; *F* (1, 37) = 0.927, *p* = 0.437].

#### Oxytocin receptor knockdown in the NAc, ACC, and BLA induces region specific effects on local oxytocin levels

There was an interaction effect between OXTR knockdown and SPA test in the ACC [Figure 3E; *F* (1, 30) = 7.120, *p* = 0.012] and the BLA [Figure 3E; *F* (1, 30) = 11.668, *p* = 0.002] where OXTR receptor knockdown in these regions significantly reduced PVN oxytocin levels in the groups not exposed to the SPA test relative to their control counterpart, and no effects on SPA test across conditions. OXTR knockdown in the NAc did not affect oxytocin content in the PVN [Figure 3E; *F* (1, 32) = 2.708, *p* = 0.110]. In addition, There were no differences in oxytocin levels across conditions in subjects injected in the NAc [Figure 3F; H (3) = 5.219, *p* = 0.156], and the ACC [Figure 3F; H (3) = 6.496, *p* = 0.090]. However, voles experiencing the SPA test had elevated oxytocin levels in the BLA regardless of viral treatment [Figure 3F; H (3) = 18.985, *p* ≤ 0.001].

#### Oxytocin receptor knockdown in the NAc, ACC, and BLA induces region specific effects on MAPK signaling

**Figure 4:**
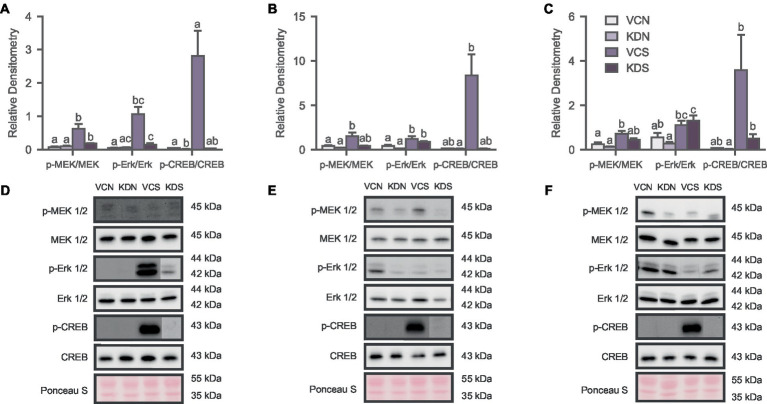
Oxytocin receptor knockdown decreases basal CREB activity in the NAc. Activity of the signaling pathway expressed as a ratio of the phosphorylated and non-phosphorylated densitometry of the markers for **(A)** NAc; **(B)** ACC; and **(C)** BLA. Letters above bars significantly differ from values with no shared letters within each marker (*N* = 8–12 per condition). Representative blots for MAPK markers of **(D)** NAc Injected groups; **(E)** ACC injected groups; and **(F)** BLA injected groups. VCN, viral controls not exposed to the SPA test; KDN, knockdown subjects not exposed to the SPA test; VCS, viral controls exposed to the SPA test; KDS, knockdown subjects exposed to the SPA test.

We analyzed the phosphorylation state of the MAPK pathway to determine whether OXTR downregulation in the NAc, ACC, and BLA drives the changes previously observed in defeated animals. We observed significant differences across markers in the NAc [Figure 4A; MEK 1/2, H (3) = 15.918, *p* = 0.001; Erk 1/2, H (3) = 9.523, *p* = 0.023; CREB, H (3) = 12.026, *p* = 0.007]; the ACC [Figure 4B; MEK 1/2, H (3) = 10.224, *p* = 0.017; Erk 1/2, H (3) = 12.753, *p* = 0.005], and the BLA [Figure 4C; MEK 1/2, H (3) = 9.713, *p* = 0.021; Erk 1/2, H (3) = 12.071, *p* = 0.007; CREB, H (3) = 8.421, *p* = 0.038], mostly driven by the exposure to the SPA test. Specifically, post-hoc analysis revealed that the basal activity of the signaling pathway remained unchanged after OXTR knockdown across markers in the ACC and the BLA (Figures 4A,B). However, CREB phosphorylation decreased in the NAc of the knockdown group not exposed to SPA relative to its control counterpart (Figure 4A). Notably, SPA testing increased the phosphorylation of the MAPK pathway across regions and conditions (Figure 4). These results indicate that the basal activity state of the MAPK signaling pathway decreases in the NAc after OXTR knockdown and that the behavioral test increases activity of the MAPK pathway regardless of treatment.

## Discussion

In the present study, we found that defeat leads to social avoidance in female prairie voles and OXTR downregulation in the NAc, as previously reported (Hale et al., 2021). Similarly, OXTR knockdown in the NAc, ACC, and BLA of stress-naïve females prevents social preference. Experiment 2 revealed that OXTR knockdown dampens MAPK basal activity in the NAc. However, experiment 1 showed that defeat leads to a complex region-, stress- and stimuli-dependent MAPK response, suggesting the recruitment of oxytocin signaling and other systems affecting this pathway. It is worth noting that our experimental design did not allow for the isolation of OXTR-expressing cells to assess cell-type-specific changes in the signaling cascade. As such, these results on MAPK reflect the net response from several cell types within each region, for which future studies should consider specific analysis of isolated OXTR-expressing cells to add to these results. Finally, defeat and OXTR knockdown induced changes in local oxytocin levels in the ACC, BLA, and PVN, suggesting a compensatory response from the oxytocin system to the insults affecting receptor expression.

### Regulation of social avoidance

Oxytocin receptor signaling is required for the expression of social affiliation in mesolimbic regions of the brain. For instance, overexpression of OXTR in the NAc of female prairie voles facilitates alloparenting and pair bond formation (Keebaugh and Young, 2011). Conversely, administering an oxytocin antagonist in the NAc during cohabitation prevents partner preference in female prairie voles (Young et al., 2001), and OXTR knockdown in the NAc reduces alloparental behavior and partner preference (Keebaugh et al., 2015). Similarly, *Oxtr* knockout in the NAc shell leads to pup abandonment phenotype in post-parturient mice, affecting the onset of maternal behavior in this species (Witchey et al., 2024). Social stress can also affect sociability and OXTR expression in the brain. Fourteen consecutive days of defeat conditioning leads to anxiety-like behaviors and downregulation of OXTR in the ACC of Mandarin voles (Li et al., 2020). *Oxtr* expression is reduced after 3 days of defeat conditioning in female not male California mice (Williams et al., 2020). Similarly, a three-day social defeat conditioning in prairie voles induces anxiety-like behaviors, social avoidance, and progressive downregulation of OXTR in the NAc, ACC, and BLA over an eight-week period (Tickerhoof et al., 2020; Hale et al., 2021). We observed that social defeat significantly decreased social investigation, reproducing the avoidant phenotype previously described in this model (Tickerhoof et al., 2020; Hale et al., 2021). However, we found that OXTR binding decreased only in the NAc, as receptor binding in the BLA and ACC was not affected as initially reported (Hale et al., 2021). One potential reason for this difference is that we reduced the intensity of the defeat experience in this study compared to our previous modeling by introducing a maximal attack criterion (i.e., seven attacks). Thus, these results may suggest that the OXTR sensitivity to social defeat is region-specific and depends on the extent of stress experienced.

It has been previously reported that stress response in female prairie voles depends on stress intensity and predictability of the stressor, where less intense and more predictable experiences result in modest behavioral and physiological responses to stress (Smith et al., 2013). Defeat conditioning previously studied in our lab involved 15-min sessions of physical resident-intruder interactions followed by 45 min of sensory exposure to the olfactory cues of the aggressor for three consecutive days. The validation of this model showed that defeated animals received 15 offensive attacks on average during that time window (Tickerhoof et al., 2020), resulting in progressive and robust downregulation of OXTR in the NAc, ACC, and BLA of female prairie voles (Hale et al., 2021). In the current study, animals were exposed to physical sessions of 7 attacks or 15 min, whatever happened first, followed by sensory exposure to complete 1 h of behavioral testing for three consecutive days. This suggests less aggressive and more predictable stress experiences, with subjects receiving up to 7 attacks per defeat session. While the mechanistic aspects of different sensitivities of these regions to the intensity of stress are still elusive, the behavioral effects of defeat were consistent with the dysregulation of OXTR in the NAc as reported in other species (Wang L. et al., 2018; Williams et al., 2020; Hou et al., 2021), revealing the relevance and high sensitivity of this region to social stress and social reward processing.

Results from Experiment 2 further confirm the role of OXTR density in SPA behavior. Animals injected with the viral control showed social preference, which was ablated after OXTR knockdown in the NAc, BLA, and ACC. One limitation to note is that control subjects injected in each region were pooled to reduce the number of animals. While this means that there is not an adequate number of controls per injection site, all regions were represented in the viral control condition with no significant effects of injection site in SPA behavior. The behavioral results observed after OXTR knockdown in these regions closely resemble the avoidant phenotype of defeated animals in Experiment 1 and address previous reports of social deficits induced by local OXTR knockdown in female prairie voles (Keebaugh et al., 2015). Affiliative behaviors in rodent animal models have also been positively correlated with endogenous differences in OXTR expression. For instance, prairie voles have a higher density of OXTR in the NAc than rats, mice, and meadow voles; species where social behaviors such as alloparenting and monogamy are absent or not spontaneous as in prairie voles (Olazábal and Young, 2006). Similarly, there is a higher density of OXTR in the BLA (Insel and Shapiro, 1992) and medial prefrontal cortex (mPFC) of prairie voles compared to the mountain vole, a polygamous and relatively less social species (Insel and Shapiro, 1992; Smeltzer et al., 2006). Further, social deficits have been reported in OXTR knockout mice and prairie voles (Pobbe et al., 2012; Horie et al., 2019). These studies suggest that OXTR density and activity in the NAc, BLA, and ACC can regulate the expression of affiliative behaviors and highlight these regions as important mediators of social preference in female prairie voles. However, further studies are required to explore the apparent region-specific sensitivity to defeat observed in Experiment 1 in light of the well-documented involvement of the limbic system in social cognition and stress response (Kent and Rauch, 2003; Roozendaal et al., 2009).

### Regulation of oxytocin local availability

Stress conditioning and social experience increase oxytocin release in selected brain regions. For instance, social defeat significantly increases oxytocin release in the mediolateral septum of male rats (Ebner et al., 2000). Immobilization stress and subsequent social interaction increase local oxytocin release in the PVN of female prairie voles (Smith and Wang, 2014). In confirmation, we observed region-specific effects on local oxytocin availability, where the behavioral test increased oxytocin levels in the ACC of defeated animals, defeat alone trends to increase oxytocin in the BLA, and oxytocin levels in the NAc remained unchanged throughout conditions. Considering the effects of oxytocin in anxiolysis and affiliative behaviors in mesolimbic regions (Blume et al., 2008; Yoshida et al., 2009; Smith et al., 2016; Wang L. et al., 2018), increased oxytocin in ACC and BLA might be a compensatory mechanism of the oxytocin system to mitigate the behavioral effects of social defeat. It is worth noting that while the OXTR binding in the ACC and the BLA remained unchanged after defeat, both regions showed increased oxytocin levels in the stressed subjects after the social test. Sustained and persistent activation of G-protein coupled receptors (GPCR) leads to internalization and later degradation of the receptor. Therefore, increased oxytocin levels in the ACC and BLA of defeated groups may suggest future ligand-mediated and socially driven downregulation of the receptor in these regions.

Previous studies have shown that stress decreases oxytocin immunoreactivity in the PVN. For instance, neonatal isolation and paternal deprivation induce anxiety-like behaviors and reduce the number of oxytocin immunoreactive neurons in the PVN of mandarin voles (Wei et al., 2013; He et al., 2019). We observed similar results where OXTR knockdown in the ACC and BLA decreased PVN oxytocin levels in the groups not exposed to the SPA test. As the PVN is the primary source of oxytocin in the brain (Jurek and Neumann, 2018), these results suggest a signaling feedback in the oxytocin system to reduce the production of the neuropeptide to compensate the downregulation of OXTRs. Conversely, Experiment 1 showed that the oxytocin content in the PVN remained unchanged after defeat conditioning and the behavioral test. Previous studies have shown that c-fos activity increases after defeat in the PVN and the medioventral bed nucleus of the stria terminalis (BNSTmv) of female California mice, but only *Oxt* mRNA levels increases in the BNSTmv (Steinman et al., 2016). Similarly, defeat-induced oxytocin production in the BNST promotes social avoidance and vigilance (Duque-Wilckens et al., 2020); while oxytocin antagonism in this region recover the social deficits induced by defeat (Duque-Wilckens et al., 2018). These studies suggest that the behavioral effects observed in defeated female prairie voles might also be regulated by extrahypothalamic oxytocin regions to mediate the social deficits observed in this model, but this warrants further research.

### Regulation of the MAPK pathway

The MAPK pathway is associated with stress regulation and the anxiolytic effects of oxytocin (Meller et al., 2003; Pardon et al., 2005; Blume et al., 2008). For instance, administration of oxytocin in the PVN increases the phosphorylation state of MAPK signaling proteins (Blume et al., 2008; Jurek et al., 2012). Similarly, oxytocin modulates anxiety-like behaviors through PKC-ERK signaling in the ACC of male and female mice (Li et al., 2021). This current data shows for the first time that: (a) social defeat has region-specific effects on MAPK activity; (b) MAPK is responsive to a social challenge (i.e., the SPA test) in a stress-dependent manner and, (c) OXTR knockdown in female prairie voles decreases basal CREB phosphorylation in the NAc.

Results on the activation state of the MAPK cascade after defeat showed hypoactivity of the cascade in the NAc, as observed in the Erk 1/2 phosphorylation ratio, followed by increased activity after the SPA test. Additionally, oxytocin levels in the NAc were unchanged, suggesting that these changes in the signaling pathway were driven by the decrease in OXTR availability, at least in the groups not exposed to the SPA test. Decreased MAPK activity in the NAc has also been reported in male mice conditioned with repeated swim stress (Bruchas et al., 2008). Further, defeat decreases *p-*CREB levels in the NAc of male Long-Evans rats with no changes in the activity of Erk 1/2 (Yap et al., 2015). These studies suggest that social defeat decreases OXTR and the basal activation state of the MAPK pathway in the NAc, which might later affect the activation of final downstream effectors to mediate the behavioral affects observed after stress conditioning.

CREB is one of the canonical final effectors of the MAPK signaling pathway. This transcription factor facilitates long-term potentiation (LTP) and conditioned memory by promoting the transcription of proteins that mediate neuronal plasticity (Impey et al., 1999). Changes in CREB activity within the NAc are associated with changes in stress response and behavior. For instance, social isolation decreases CREB activity in the NAc shell and leads to anxiety-like behaviors, both recovered after antidepressant treatment (Wallace et al., 2009). Similarly, increasing CREB activity in the same region increases the behavioral response to anxiogenic, aversive, and nociceptive stimuli (Barrot et al., 2002). These studies support our findings on the NAc, where defeat leads to hypoactivity of the signaling pathway in the basal state and increased MAPK phosphorylation and social avoidance in the SPA test. Experiment 2 showed that the basal state of the MAPK pathway decreased after OXTR knockdown in the NAc reducing CREB phosphorylation. Conversely, MAPK response to the behavioral test increased regardless of condition in p-MEK/MEK ratio. While these results resemble the effects of defeat in the signaling pathway, it is worth noting that CREB and the MAPK pathway are also regulated by systems previously reported to respond to social defeat. For instance, social defeat elicits increased dopamine release in the NAc (Tidey and Miczek, 1996; Nemets et al., 2022), and dopamine receptors are known to affect MAPK and CREB phosphorylation (Wang H. et al., 2018). Therefore, future studies should explore cell-specific changes in the NAc driving the observed changes in these markers.

The effects of the SPA test on the phosphorylation of MAPK in the ACC and the BLA knockdown groups were similar to those observed in the NAc. However, the basal activity of the signaling pathway remained unchanged after OXTR knockdown, with no changes in local oxytocin levels within these regions. Further, after exposure to the SPA test, the BLA knockdown group showed increased Erk 1/2 and CREB phosphorylation. While experiment 1 showed no changes in OXTR expression in the ACC and the BLA resulting from defeat, the MAPK was hyperactive in both regions, as CREB activity was higher in the basal state of defeated subjects relative to controls. Interestingly, defeat increased oxytocin levels in the ACC and the BLA, suggesting that these changes in the basal state are ligand-driven. However, exposure to the behavioral test had region-specific changes as MAPK phosphorylation remained unchanged in the BLA and decreased in the ACC of defeated animals. These divergent results suggest that oxytocin may orchestrate changes in MAPK phosphorylation with other neuronal modulators, at least in the socially evoked state, or that oxytocin signaling in the ACC and the BLA may regulate avoidant behavior through other mechanisms within these regions.

Oxytocin is known to regulate synaptic transmission by modulating the activity of ion channels in the membrane (Bakos et al., 2018). This property has been associated with the regulation of anxiety-like behaviors in the ACC and the amygdala. For instance, the anxiolytic effect of oxytocin has been correlated with its ability to increase depolarization of interneurons in the ACC (Li et al., 2021). Similarly, oxytocin increases spontaneous inhibitory transmission in the lateral amygdala, blocking long-term potentiation of cortical inputs into this region (Crane et al., 2020). Experiment 2 showed that OXTR knockdown leads to social avoidance with modest changes in MAPK activity. This suggests that OXTR knockdown mediated social avoidance through the modulation of synaptic transmission rather than changes in the signaling pathway. This is particularly interesting as the amygdala and the cortex share reciprocal projections (Ko, 2017), and stress conditioning impairs their connectivity (Ishikawa et al., 2015). Therefore, future studies should explore oxytocin effects on local neuronal activity and functional connectivity between the ACC and the BLA to determine the molecular mechanisms by which OXTR mediates social avoidance in female prairie voles in these regions.

In conclusion, our results demonstrate that social defeat and OXTR knockdown in the NAc, ACC, and BLA of female prairie voles induce social avoidance and dysregulation of the MAPK pathway in a region- and stimuli-dependent manner. To the best of our knowledge, this is the first research that studies the resting state of the MAPK cascade and oxytocin system in control and defeated animals and compares it with the effects of a behavioral test involving social novelty. Some of the divergent effects between the activity of the signaling pathway and oxytocin dysregulation reported in this research provide a ground floor for future studies of the neuropeptide and its mechanistic relationship with other systems impacted in social defeat. Further, our results prompt research on oxytocin-mediated neuronal plasticity in mesolimbic regions, as this neuropeptide has been reportedly associated with tuning the connectivity of regions involved in stress response (Baumgartner et al., 2008; Sripada et al., 2013; Tan et al., 2019). These studies would expand the knowledge of the documented oxytocin dysregulation and contribute to the biological and behavioral characterization of social defeat in female prairie voles as a model of SAD in humans.

## Data availability statement

The raw data supporting the conclusions of this article will be made available by the authors, without undue reservation.

## Ethics statement

The animal study was approved by Animal Care and Use Committee of The University of Kansas. The study was conducted in accordance with the local legislation and institutional requirements.

## Author contributions

LN-M: Conceptualization, Data curation, Formal analysis, Funding acquisition, Investigation, Methodology, Project administration, Validation, Visualization, Writing – original draft, Writing – review & editing. AB: Funding acquisition, Methodology, Resources, Validation, Writing – review & editing. LY: Funding acquisition, Methodology, Resources, Supervision, Validation, Writing – review & editing. ML: Formal analysis, Supervision, Writing – review & editing. AS: Conceptualization, Formal analysis, Funding acquisition, Methodology, Project administration, Supervision, Visualization, Writing – review & editing.
