# Supplementary material for: Limbic oxytocin receptor expression alters molecular signaling and social avoidance behavior in female prairie voles (Microtus ochrogaster)
Source: Front Neurosci. 2024 Jul 16;18:1409316. doi: 10.3389/fnins.2024.1409316 (PMC11286410; doi:10.3389/fnins.2024.1409316)
Supplement: Supplementary file 15 [file Data_Sheet_1.DOCX]

Supplementary Material

**Lina K. Nerio Morales M.Sc., Arjen J. Boender Ph.D., Larry J. Young Ph.D, Marisol R. Lamprea Ph.D., Adam S. Smith Ph.D.***

*** Correspondence:** Corresponding Author: adamsmith@ku.edu

**1. Supplementary Figures**

**Supplementary Figure 1.** Experiment 1 - Western blot membrane with representative blots for MEK1/2.

**Supplementary Figure 2.** Experiment 1 - Western blot membrane with representative blots for p-MEK1/2.

**Supplementary Figure 3.** Experiment 1 - Western blot membrane with representative blots for Erk 1/2.

**Supplementary Figure 4.** Experiment 1 - Western blot membrane with representative blots for p-Erk 1/2.

**Supplementary Figure 5.** Experiment 1 - Western blot membrane with representative blots for CREB.

**Supplementary Figure 6.** Experiment 1 - Western blot membrane with representative blots for p-CREB.

**Supplementary Figure 7.** Experiment 1 - Western blot membrane with representative blots for Ponceau S staining.

**Supplementary Figure 8.** Experiment 2 - Western blot membrane with representative blots for MEK1/2.

**Supplementary Figure 9.** Experiment 2 - Western blot membrane with representative blots for p-MEK1/2.

**Supplementary Figure 10.** Experiment 2 - Western blot membrane with representative blots for Erk 1/2.

**Supplementary Figure 11.** Experiment 2 - Western blot membrane with representative blots for p-Erk 1/2.

**Supplementary Figure 12.** Experiment 2 - Western blot membrane with representative blots for CREB.

**Supplementary Figure 13.** Experiment 2 - Western blot membrane with representative blots for p-CREB.

**Supplementary Figure 14.** Experiment 2 - Western blot membrane with representative blots for Ponceau S staining.

**2. Supplementary Tables**

**Supplementary Table 1.** Stereotactic coordinates. *NAc- Nucleus accumbens, ACC- Anterior cingulate cortex, BLA- Basal lateral amygdala*

| **Region** | **Medial-lateral (mm)** | **Dorsal-ventral (mm)** | **Anterior-posterior (mm)** | **Adjustment^1^** |
| --- | --- | --- | --- | --- |
| NAc | ± 1.20 | - 5.60 | + 1.70 | AP = + 1.40 |
| ACC | ± 0.40 | - 1.95 | + 1.55 | AP = + 1.25 |
| BLA | ± 3.80 | - 5.08 | - 1.35 | AP = - 1.65 |

^1^If distance from bregma to lambda is >5.2 mm, anterior-posterior (AP) coordinate was adjusted

**Supplementary Table 2.** Brain sectioning series for Western blot, ELISA and OXTR autoradiography analysis.

| **Number of slices** | **Thickness (µm)** | **Region** | **Experiment** |
| --- | --- | --- | --- |
| 1 | 200 | NAc | Western blot, ELISA |
| 6 | 20 | NAc, ACC | Receptor Autoradiography |
| 1 | 300 | NAc, ACC | Western blot, ELISA |
| 6 | 20 | NAc, ACC | Receptor Autoradiography |
| 2 | 300 | ACC | Western blot, ELISA |
| 1 | 300 | PVN | ELISA |
| 1 | 300 | PVN, BLA | Western blot, ELISA |
| 6 | 20 | BLA | Receptor Autoradiography |
| 2 | 300 | BLA | Western blot, ELISA |

**Supplementary Table 3.** Antibodies for Western blot protein analysis of MAPK signaling pathway. *HRP- Horseradish peroxidase.*

| **Primary antibody** | **Manufacturer** | **Catalog number** | **Concentration** |
| --- | --- | --- | --- |
| Rabbit-anti-*p*-CREB | Cell Signaling | 9198 | 1:500 |
| Mouse-anti-CREB | Cell Signaling | 9104 | 1:500 |
| Rabbit-anti-*p*-Erk 1/2 | Cell Signaling | 4370 | 1:500 |
| Mouse-anti-Erk 1/2 | Cell Signaling | 9107 | 1:1000 |
| Rabbit-anti-*p*-Mek 1/2 | Cell Signaling | 9156 | 1:500 |
| Mouse-anti-Mek 1/2 | Cell Signaling | 2352 | 1:500 |
| HRP-conjugated anti-rabbit IgG | Santa Cruz | sc-516102 | 1:10000 |
| HRP-conjugated anti-mouse IgG | Santa Cruz | sc-2357 | 1:10000 |
